# Capture of microtubule plus-ends at the actin cortex promotes axophilic neuronal migration by enhancing microtubule tension in the leading process

**DOI:** 10.3389/fncel.2014.00400

**Published:** 2014-11-27

**Authors:** B. Ian Hutchins, Susan Wray

**Affiliations:** ^1^Cellular and Developmental Neurobiology Section, National Institute of Neurological Disorders and Stroke, National Institutes of HealthBethesda, MD, USA; ^2^Postdoctoral Research Associate Program, National Institute of General Medical Sciences, National Institutes of HealthBethesda, MD, USA

**Keywords:** neuronal migration, neuronal migration disorders, microtubules, IP3 receptors, EB1, super resolution microscopy, actin cytoskeleton

## Abstract

Microtubules are a critical part of neuronal polarity and leading process extension, thus microtubule movement plays an important role in neuronal migration. However, the dynamics of microtubules during the forward movement of the nucleus into the leading process (nucleokinesis) is unclear and may be dependent on the cell type and mode of migration used. In particular, little is known about cytoskeletal changes during axophilic migration, commonly used in anteroposterior neuronal migration. We recently showed that leading process actin flow in migrating GnRH neurons is controlled by a signaling cascade involving IP3 receptors, CaMKK, AMPK, and RhoA. In the present study, microtubule dynamics were examined in GnRH neurons. Failure of the migration of these cells leads to the neuroendocrine disorder Kallmann Syndrome. Microtubules translocated forward along the leading process shaft during migration, but reversed direction and moved toward the nucleus when migration stalled. Blocking calcium release through IP3 receptors halted migration and induced the same reversal of microtubule translocation, while blocking cortical actin flow prevented microtubules from translocating toward the distal leading process. Super-resolution imaging revealed that microtubule plus-end tips are captured at the actin cortex through calcium-dependent mechanisms. This work shows that cortical actin flow draws the microtubule network forward through calcium-dependent capture in order to promote nucleokinesis, revealing a novel mechanism engaged by migrating neurons to facilitate movement.

## Introduction

For proper assembly of neural circuits, newly born neurons must migrate from their place of origin to their final location. Neuronal migration is commonly classified by the pathway the cells use, e.g., radial, tangential, or anteroposterior—anatomically indicating orientation to the cortex (Marín et al., 2010). However, neurons use many modes of migration within these categories. Some features are common to multiple populations of neurons such as saltatory locomotion occurring in radially migrating cortical neurons as well as in the axophilic migration of GnRH neurons (Nadarajah et al., 2001; Casoni et al., 2012). Similar features between different types of migrating neurons indicate that conserved movement mechanisms exist. Yet, certain aspects, such as the basic mechanisms underlying movement of cells during migration are clearly variable. These mechanisms include locomotion and nucleokinesis (Schaar and McConnell, 2005; Tsai and Gleeson, 2005), rapid spring-like somal translocation (Nadarajah et al., 2001), iterative extension and retraction of leading process branches (Martini et al., 2009), a highly branched “climbing mode” for pathfinding (Kitazawa et al., 2014) or multipolar migration (Tabata and Nakajima, 2003; Falnikar et al., 2013). These different mechanisms of migration often exhibit major alterations in the actin cytoskeleton (Solecki et al., 2009; Asada and Sanada, 2010; He et al., 2010; Martini and Valdeolmillos, 2010; Hutchins et al., 2013). Actin dynamics can promote neuronal migration by propulsive contractions at the cell rear (Martini and Valdeolmillos, 2010; Steinecke et al., 2014), through leading process actin dynamics away from the soma (Solecki et al., 2009; He et al., 2010), or via both mechanisms in tandem (Hutchins et al., 2013). However, microtubule forces, together with actin are most likely responsible for generating the sequential steps of nuclear translocation and neuronal cell migration (Pollard and Borisy, 2003; Tolić-Nørrelykke, 2010; Lysko et al., 2014). Microtubules surround the nucleus. In the leading process, extended bundles of microtubules emanating from the centrosome define the direction of movement. Live-cell imaging data from mouse cerebellar granule cells showed that movement of nucleus and centrosome occur independently (Umeshima et al., 2007). These data suggest the existence of a pathway that may depend on a decentralized (i.e., away from the centrosome) microtubule organization and/or an interaction with actin cytoskeleton (Schaar and McConnell, 2005; Solecki et al., 2009).

The present study investigates the role of microtubules in neurons exhibiting axophilic anteroposterior migration, GnRH (gonadotropin releasing hormone 1-expressing) neurons. Recent work revealed that IP3 receptors promote nucleokinesis in these cells, signaling through CaMKK, AMPK, and RhoA, to engage cortical actin flow toward the distal leading process (Hutchins et al., 2013). Here, using the same model system, we show that microtubule linkage to the dynamic cortical actin in the leading process shaft transmit forces critical for nucleokinesis.

## Materials and methods

### Nasal explants

All procedures were approved by NINDS ACUC and performed according to NIH guidelines. Explants were generated from E11.5 embryos of either gender as previously described (Klenke and Taylor-Burds, 2012). Explants were incubated at 37°C in defined serum-free medium (SFM) in 5% CO2. Pharmacological treatments included 75 μM 2-APB (Tocris Bioscience), 1 μM nocodazole (Tocris Bioscience), and Concanavalin A (10 μg/mL, Vector Labs).

### Confocal microscopy

Images were acquired using a Nikon TE200 microscope with a CSU10 spinning disk confocal (Yokogawa, Tokyo, Japan) and Hamumatsu ImagEM C9100-13 EMCCD camera (Hamumatsu, Hamumatsu, Japan) with a 60× objective (Nikon, Melville, NY) for microtubule imaging or Retiga SRV (Qimaging, Surrey, BC, Canada) with a 20× ELWD for DIC imaging.

### Super-resolution imaging

Images were acquired using a Leica CW STED Confocal microscope (stimulated emission depletion) (Klar et al., 2000) with a 100× oil immersion objective (Leica). Images were over-sampled by a factor of ~2.4 with a pixel size of 37.5 nm. Samples were fixed in 4% formaldehyde in PHEM buffer at 37°C for 1 h and prepared for two-color STED microscopy with Atto 425 phalloidin (5 units/mL, equivalent to 165 nM, Sigma), and EB1 primary antibodies (1:100, BD) labeled with Oregon Green 488 secondary antibodies (1:1000, Life Technologies). Cells were also immunostained for GnRH (SW-1, 1:3000) (Hutchins et al., 2013) and labeled with Alexa Fluor 647 (1:1000, Life Technologies); this channel was imaged with conventional microscopy immediately prior to STED imaging, which bleached this fluorophore.

### Microtubule imaging

Microtubules were labeled by bath application of 150 nM TubulinTracker Green (Life Technologies) for up to 25 min. Six micrometer z-stacks at 1.5 μm intervals were acquired every 30 s for imaging sessions lasting up to 20 min. During axophilic migration, GnRH neurons are closely apposed to olfactory sensory axons; measurements of microtubule dynamics were carefully taken to avoid fluorescence signal from intersecting pathway axons. Z-stacks were flattened for image analysis. Microtubules were manually tracked. This method was validated with automated cross-correlation measurement from the same cells (TRACKER ImageJ plugin, Olivier Cardoso, Paris Diderot University, set to 9 × 9 pixel regions and 3-pixel correlation size) (Hutchins et al., 2013). In the 11 control cells that were cross-validated, manual and automated tracking of microtubules over the entire imaging session yielded a striking correspondence (*R*^2^ = 0.7840, simple linear regression). Nucleus centroids were tracked to calculate migration rates. GnRH neurons monitored by fluorescence imaging showed similar rates of movement (23.92 ± 6.46 μm/h) as unlabeled cells monitored by DIC imaging (23.27 ± 1.42 μm/h). To ensure that microtubule and nuclear movement occurred at the same time, movies were segmented into 2-min frames and movement compared within those frames. Microtubule/soma convergence was measured as the decrease over time of the distance between the edge of the soma and leading process microtubule bundles. Negative convergence indicates that microtubules are separating away from the edge of the soma.

### Statistics

Statistics were performed in Prism 5 (GraphPad, La Jolla, CA) or R (R-Project; for 3D scatter plot and multiple regression). Unless otherwise noted, n is the number of cells and N is the number of explants. Model II linear regression was used to analyze cytoskeletal dynamics to account for measurement error in both the dependent and independent axes (Hutchins et al., 2013). This total least squares regression minimizes the sum of squared distances from the points to the regression line. Pearson's correlation coefficient *r* is given as a measure of effect size for these analyses.

Residuals analysis is performed to examine the contribution of a second parameter on a measured variable. This analysis was used to determine whether microtubule/soma convergence contributed to migration rate after removing the effects of forward microtubule movement. Residual soma speeds were evaluated with Model II linear regression as stated above.

## Results

**Figure 1:**
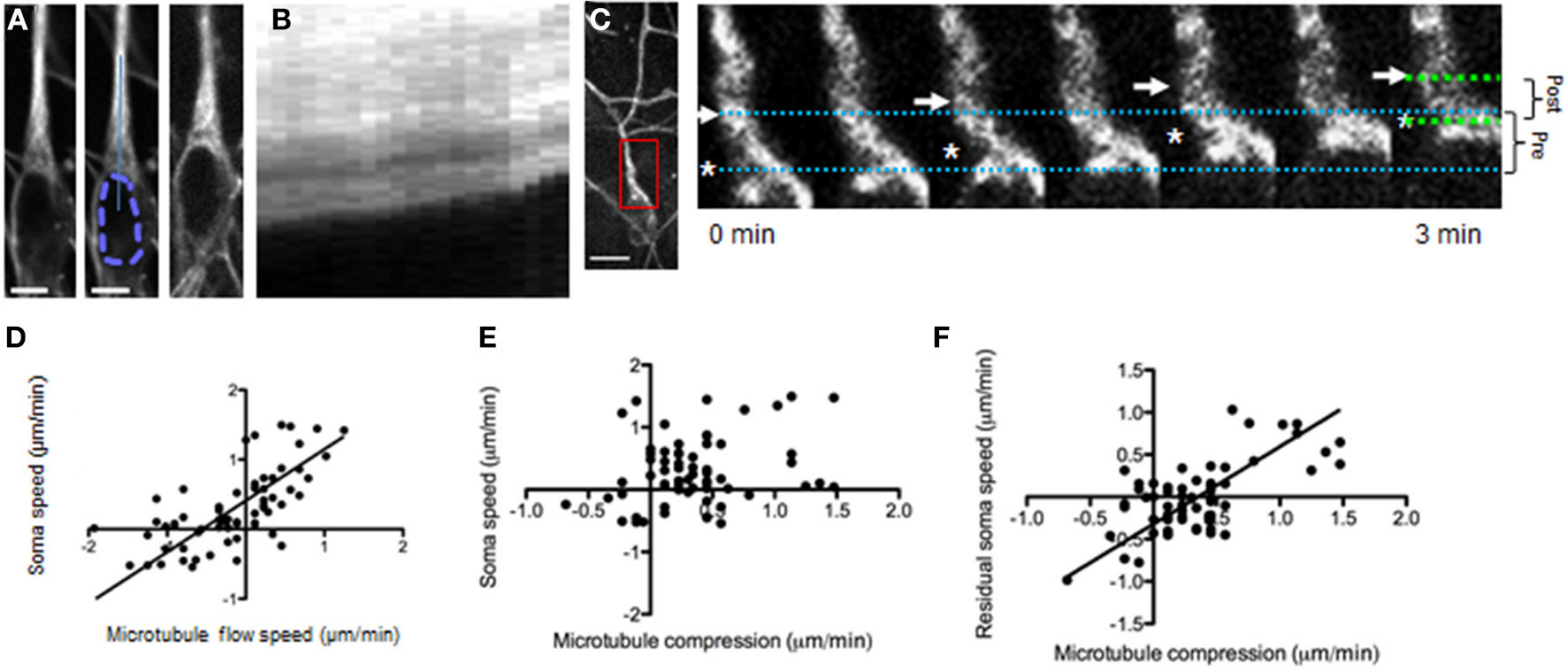
**Microtubule dynamics during neuronal migration**. **(A)** Microtubules in a migrating GnRH neuron (raw fluorescence left, scale bar, 5 μm; mid, nucleus outlined with dashed line; solid line indicates region for generating kymograph; image of the same cell at the end of the imaging session, right). **(B)** The microtubules translocate forward during neuronal migration (kymograph, duration 10 min). (**C**, left) Lower magnification view of fluorescent microtubule staining in a migrating GnRH neuron. Inset, region of higher-magnification region shown in pseudocolored time-lapse images (right). Scale bar, 10 μm. (**C**, right) Simultaneous forward microtubule translocation (arrow) and convergence with the soma (asterisk). Dotted lines show the distance between the soma edge and microtubule bundles at the beginning (blue) and end (green) of the imaging session; brackets (right) summarize the change in these distances from beginning (pre, 3.9 μm) to end (post, 2.7 μm). **(D–F)** Frame-by-frame analysis was performed (*n* = 65) on 2-min frames from 12 neurons (*N* = 9 explants). **(D)** Forward translocation of microtubules vs. soma speed within 2-min time frames (*p* < 0.0001, linear regression, *r* = 0.68). **(E)** Microtubule/soma convergence vs. soma speed shows only a weak relationship (*p* = 0.032, linear regression, *r* = 0.27). **(F)** Microtubule/soma convergence accounts for much of the residual soma speed after subtracting the effect of microtubule translocation rates (*p* < 0.0001, linear regression, *r* = 0.68).

Different roles for microtubules in neuronal migration have been described including pulling the nucleus along the leading process toward the growth cone (Tsai et al., 2007; Asada and Sanada, 2010) and forming a barrier to nucleokinesis (He et al., 2010; Martini and Valdeolmillos, 2010; Falnikar et al., 2011). Observing microtubule dynamics with TubulinTracker Green showed that leading process microtubules translocated toward the growth cone during migration (Figures 1A–C). In addition, a strong relationship between the speed and direction of microtubule translocation with movement of the cell body was found (Figure 1D).

The relationship between simultaneous translocation of both microtubules and the nucleus toward the growth cone appears consistent with the proposed role of microtubules pulling the nucleus toward the growth cone (Asada and Sanada, 2010). However, in our experiments, single cell analysis revealed that the soma frequently advanced faster than the microtubules, corresponding to compression of the soma against microtubule bundles located immediately adjacent to the nucleus (Figure 1C). This soma/microtubule compression was measured as the speed at which the front edge of the soma and the microtubule bundle converged. This observation could be evidence of a microtubule barrier in front of the nucleus. However, only a weak first-order relationship between soma/microtubule compression and migration rates was found (Figure 1E).

One possibility is that forces causing soma/microtubule compression add to the influence of microtubule translocation described above. In this case compression should be compared to the residual migration rate (the migration rate left over after subtracting out the influence of microtubule translocation) to detect an additive contribution. To this end, a residuals analysis was performed (Hutchins et al., 2013). Residuals analysis subtracts the influence of the first independent variable (microtubule translocation rates) from the dependent variable (migration rate), giving a “residual” migration rate that can be compared to a new independent variable (soma/microtubule compression). This analysis revealed a strong correlation between soma/microtubule compression and the residual migration rate (Figure 1F). How well do these two measures combine to predict migration rates? The relationship between microtubule speed, soma/microtubule compression and soma speed are shown in a 3D scatterplot with a best-fit plane (Movie 1 shows the 3D scatterplot). These data indicate that microtubule translocation and soma/microtubule compression strongly predict movement of the nucleus when taken together (multiple regression *R*^2^ = 0.7696).

**Figure 2:**
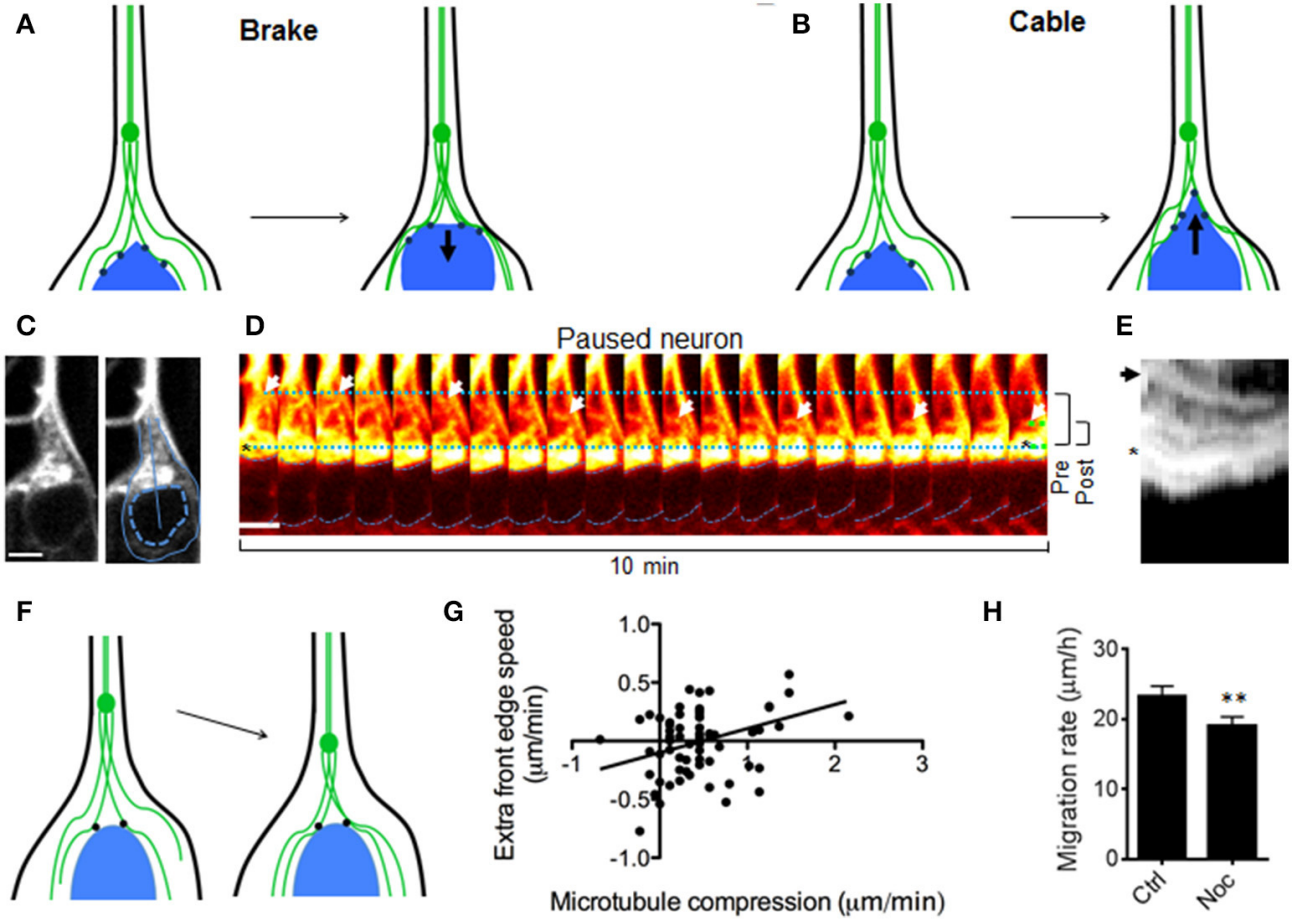
**Models to explain convergence between nucleus and leading process microtubules**. **(A)** Testable model 1 (“Brake”): Microtubules act as a brake. Nucleokinesis is due to pushing forces from behind that cause the nucleus to “crash” against leading process microtubules. Leading process microtubules (green) form a barrier that slows (resistive force shown as arrow) the front edge of the nucleus (blue) as these compress together. In this model microtubule convergence and excess speed of the front edge are inversely correlated, and this convergence only occurs during nucleokinesis. **(B)** Testable model 2 (“Cable”): Microtubule motor proteins (black dots) draw the nucleus forward along the leading process microtubules, which can be thought of as cables or rails, as in other cell types (Zhang et al., 2009). The pulling force (arrow) from in front of the nucleus draws the front edge along microtubules faster than the center, causing elongation of the nucleus. In this model, microtubule/soma convergence and excess speed of the front edge are directly correlated, and convergence may also occur in GnRH neurons that have stalled. (**C**, left) Fluorescent staining of microtubules in a GnRH neuron that is not migrating. (**C**, right) Outlines indicate the border of the cell (solid) and nucleus (dotted), while the line shows the region measured for kymographs. Scale bar, 5 μm. **(D)** Backward microtubule translocation (arrows) in a paused neuron. Dotted lines show the distance between the soma edge and microtubule bundles at the beginning (blue) and end (green) of the imaging session; dashed blue lines denote the nucleus; brackets (right) summarize the change in these distances from beginning (pre, 6.1 μm) to end (post, 2.7 μm). Scale bar, 5 μm. **(E)** Kymograph of the region shown in **(C)**, with an asterisk and arrow corresponding to the marked regions in **(D)**; the microtubule bundle in the leading process (arrow) and the front edge of the soma (asterisk). **(F)** Schematic: During stalling, microtubules (green) reverse direction and move toward the nucleus. **(G)** Measurements of microtubule/soma convergence vs. excess speed of the front edge show a direct relationship (*p* = 0.0325, linear regression, *r* = 0.27, *n* = 65 frames from 12 neurons, *N* = 9 explants), refuting the “brake” model in **(A)** and supporting the “cable” model in **(B)**. **(H)** Acute nocodazole (at microtubule depolymerizing concentrations) reduced neuronal migration rates in DIC-imaged GnRH neurons by 22% (*p* = 0.0051, Wilcoxon matched pairs signed rank test; *n* = 107 neurons from *N* = 4 explants).

To understand the mechanism(s) underlying these observations (in particular, the soma/microtubule convergence), two pertinent models of nucleus/microtubule interactions that have been previously reported were examined (see Figures 2A,B). The microtubule brake model (Falnikar et al., 2011) proposes that cross-linked microtubules in the leading process create a barrier for nucleokinesis (Figure 2A). Soma/microtubule compression could thus result from propulsive forces from the cell rear (Martini and Valdeolmillos, 2010) as the nucleus is forced through this microtubule lattice. In this case, nucleus/microtubule compression should occur *only* when the nucleus is propelled forward. Alternatively in the second model, microtubule motor proteins can pull the nucleus along microtubule bundles, drawing the two together (Tsai et al., 2007; Zhang et al., 2009). In this scenario, nucleus/microtubule convergence should also be observed when the nucleus pauses, as microtubules are drawn backward (schematic in Figure 2B). To test these possibilities, microtubule dynamics were monitored in neurons that were spontaneously pausing. In stalled neurons, microtubules displayed rapid movement backward toward the nucleus (Figures 2C–E), as if no longer coupled to movement of the soma as they are in forward migration (see Figure 1). Robust microtubule convergence with the cell body (in this case caused by backward movement of microtubules rather than forward movement of the soma) suggested that microtubules were actively drawn toward the soma and not acting as a brake (Figure 2F).

Some predictions made by these two models of nucleus/microtubule convergence were further tested with these live imaging experiments. A microtubule brake model would predict that the leading edge of the soma might be compressed backward toward the centroid of the nucleus as it pushed against microtubules located ahead of it (Figure 2A). In contrast, if the nucleus is drawn forward along microtubules by an active mechanism (rather than by a passive collision), the nucleus edge may elongate toward the leading process as it is pulled forward along leading process microtubules (Figure 2B). These models make opposite predictions about the relationship between elongation of the leading soma edge vs. microtubules and were tested by measuring the change in distance from the nucleus centroid to its edge facing the leading process. We found that microtubule/soma convergence was directly related to soma elongation (Figure 2G), supporting an active process drawing soma and microtubules together. Studies have reported unaltered or enhanced neuronal migration when microtubules are depolymerized, consistent with a braking mechanism (Schaar and McConnell, 2005; He et al., 2010; Martini and Valdeolmillos, 2010). Conversely, if convergence of the soma and microtubules is an active process in migrating GnRH neurons (e.g., caused by motor proteins drawing the two together, rather than a passive collision caused by propulsion from the cell rear), one would predict that depolymerization of microtubules should slow, rather than accelerate migration. In our system, acute nocodazole (at microtubule depolymerizing concentrations) reduced neuronal migration rates by 22% (Figure 2H, *p* = 0.0051, Wilcoxon matched pairs signed rank test; *n* = 107 neurons from *N* = 4 explants), further supporting an active process drawing soma and microtubules together during cell migration.

**Figure 3:**
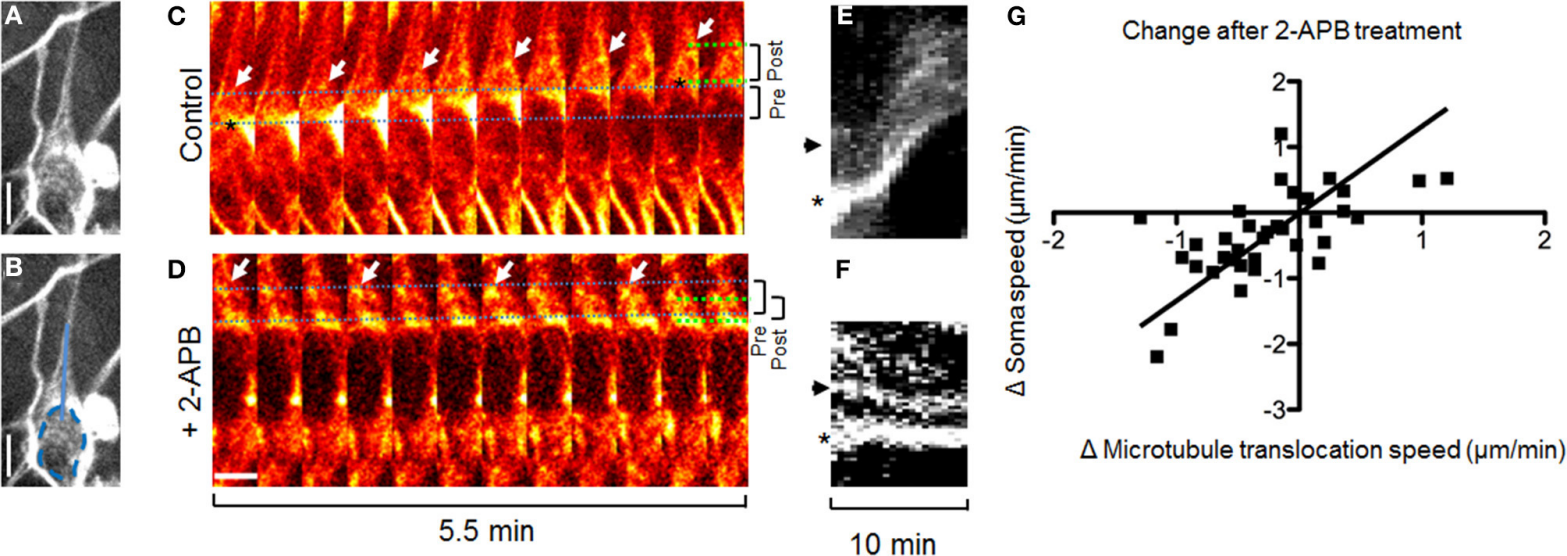
**Inhibiting IP3 receptors reverses forward translocation of microtubules**. **(A,B)** Fluorescent staining of microtubules in a migrating GnRH neuron. **(B)** Same as **(A)**, but with a dotted line *s*howing the border of the nucleus and a solid line showing the region shown for kymographs. **(C,D)** Forward translocation of microtubules (**C**, arrows) is reversed after application of 2-APB to block IP3 receptors (**D**, arrows). Dotted lines show the distance between the soma edge and microtubule bundles at the beginning (blue) and end (green) of the imaging session; brackets (right) summarize the change in these distances from beginning (pre, 3.4 μm for **C** and 3.6 μm for **D**) to end (post, 3.6 μm for **C** and 2.3 μm for **D**). Images in **(A–F)** are from the same cell. **(E,F)** Kymographs of the region shown in **(B)**, containing the microtubule bundle (arrow) and edge of the soma (asterisk) as shown in **(C,D)**. **(G)** Change in microtubule translocation rate corresponds to the change in soma speed (*n* = 35 frames from 7 neurons in *N* = 5 explants, *p* < 0.0001, *r* = 0.65, linear regression). Scale bars, 5 μm.

Cortical actin flow in the leading process promotes nucleokinesis, and thereby the migration of GnRH neurons, and is dependent on calcium release through IP3 receptors (Hutchins et al., 2013). To test whether microtubule dynamics during nucleokinesis operate using the same calcium release-dependent signaling pathway, calcium channels (IP3 receptors) were blocked with 2-APB (Li et al., 2009; Hutchins et al., 2011, 2013). TRP channels, also blocked by 2-APB, have been shown to have no effect on either spontaneous calcium activity or migration in GnRH neurons (Hutchins et al., 2013). Inhibiting calcium release through IP3 receptors reduced forward translocation of microtubules (Figure 3 and Movie 2). Notably, after application of 2-APB, microtubules were observed to reverse direction–from moving toward the growth cone to instead moving toward the stalled soma (Figures 3D,F). These data indicate that, in contrast to microtubule translocation, soma/microtubule convergence rates were unaffected by 2-APB, i.e., not dependent on calcium release (*p* = 0.23, Two-Way ANOVA, *n* = 35 frames from *N* = 5 explants). Thus, these experiments showed that movement of leading process microtubules was uncoupled from movement of the soma during calcium channel inhibition. The fact that soma/microtubule convergence remained intact during 2-APB-induced stalling (Figure 3) suggested that another mechanism(s) was being utilized. Taken together, the results indicate that microtubules translocate forward along the leading process dependent on calcium release through IP3 receptors, while simultaneously and independently, the nucleus converges with microtubule bundles.

**Figure 4:**
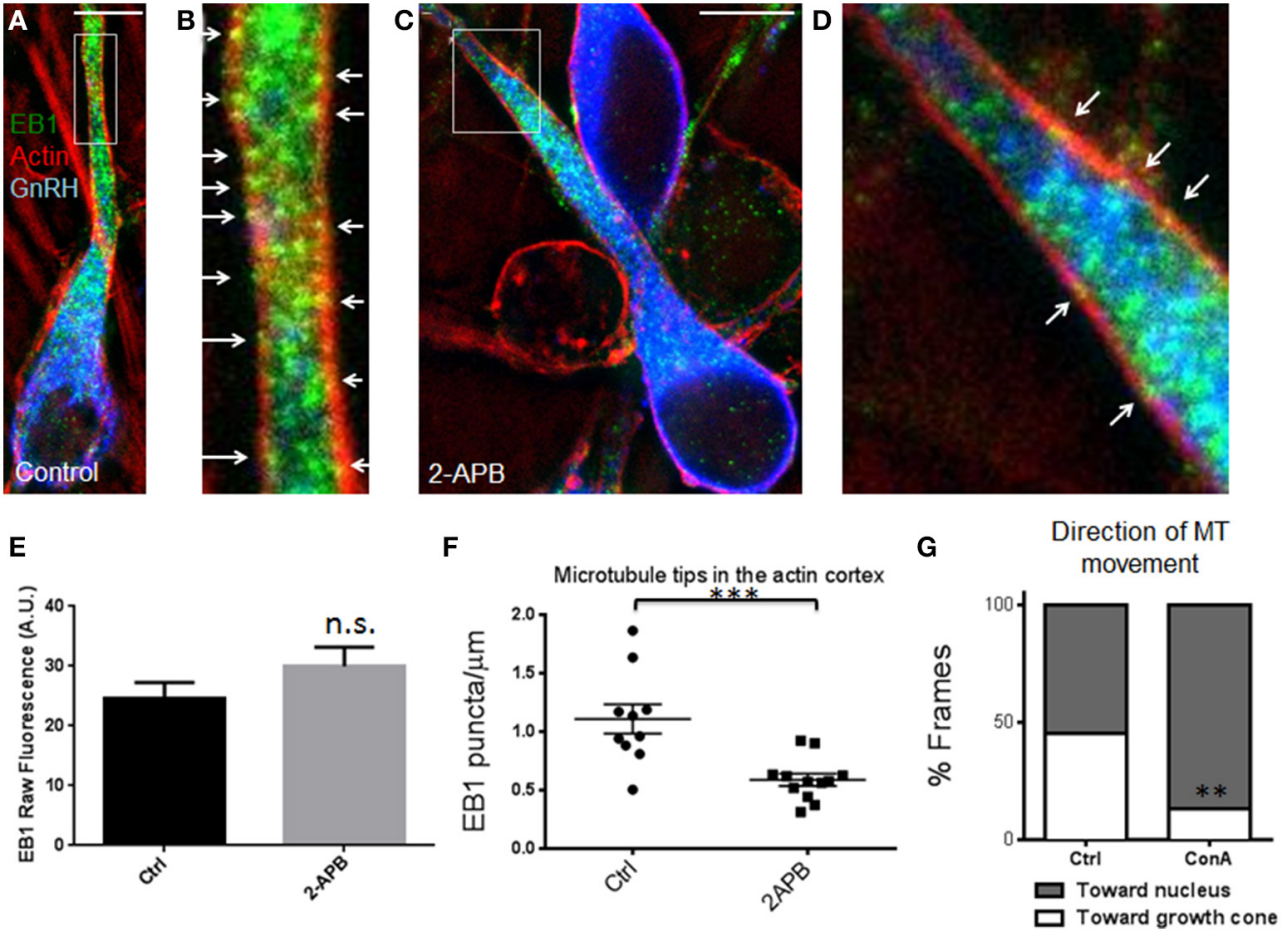
**Microtubule +TIPs lose association with cortical actin after blocking calcium release**. **(A)** Triple-staining against EB1 (green), F-actin (red), and GnRH (blue) in a control-treated cell with EB1 and F-actin imaged with STED microscopy. **(B)** Zoomed image of the box in **(A)**. Arrows indicate examples of super-resolution co-localization, showing several EB1 puncta associated with the actin cortex in the leading process. **(C,D)** Low **(C)** and high magnification (**D**, of region boxed in **C**) of triple-stained neurons treated with 2-APB. Blocking calcium release reduces the number of EB1 puncta associated with the actin cortex (arrows, **D**). Scale bars, 5 μm. **(E)** No differences were detected in raw EB1 staining fluorescence (n.s., *p* = 0.227, *t*-test, Cohen's *d* = 0.53, *n* = 10 control and 12 2-APB treated GnRH neurons). **(F)** Fewer EB1 puncta were observed in the leading process actin cortex of 2-APB treated GnRH neurons compared with vehicle controls (^***^p = 0.0006, *t*-test, Cohen's *d* = 1.75, *n* = 10 controls and 12 treated GnRH neurons, *N* = 3 explants for both conditions). **(G)** Fraction of time microtubules spent moving toward the distal growth cone was reduced by the inhibitor of cortical actin flow, Concanavalin A (*n* = 7 cells from *N* = 3 explants; ^**^p = 0.0026, Fisher's exact test).

To determine whether the forward translocation of microtubules and cortical actin flow in the leading process (Hutchins et al., 2013) are (1) directly linked or (2) simultaneously regulated by calcium release, but not mechanically coupled, two experiments were performed. In the first experiment, Concanavalin A (ConA, a cortical actin flow inhibitor) (Canman and Bement, 1997; He et al., 2010; Hutchins et al., 2013) was applied during microtubule imaging. Forward microtubule translocation was nearly abolished in the presence of ConA, while reverse translocation toward the cell body was unaffected—45% of time-lapse frames showed forward microtubule translocation in control cells (*n* = 12) vs. only 13% of frames in ConA-treated cells (*n* = 7 cells from *N* = 3 explants; *p* = 0.0026, Fisher's exact test, Figure 4). These data indicate that microtubules require both calcium release and cortical actin flow to maintain their forward movement during axophilic neuronal migration. To determine whether these structures where mechanically coupled, microtubule plus-end tracking protein EB1 was examined. Microtubule plus-end tracking proteins (+TIPs) can be coupled to actin cortex in non-neuronal cells through a protein complex including the +TIP protein EB1 (Wen et al., 2004). If this protein complex also contributes to the phenomena observed here, then EB1 puncta should co-localize with the actin cortex in migratory GnRH neurons in a calcium release-dependent manner. To test for microtubule capture at cortical actin in GnRH neurons, microtubule plus-end locations were labeled with antibodies to EB1 (Jaworski et al., 2009). Conventional microscopy was unable to provide the resolution necessary to test this hypothesis, so super-resolution STED microscopy was used. Two-color super-resolution imaging of EB1 and phalloidin revealed many EB1 puncta embedded in the actin cortex in control neurons (Figures 4A–F). To determine whether manipulating calcium release through IP3 receptors altered microtubule capture at the actin cortex, 2-APB was applied. No differences in raw EB1 fluorescence signal were detected in the leading process shaft of vehicle and 2-APB treated cells (Figure 4E, 24.6 ± 2.6 arbitrary fluorescence units in vehicle treated cells vs. 30.0 ± 3.2 in 2-APB treated cells; *p* = 0.227, *t*-test, Cohen's *d* = 0.53), suggesting that treatment with 2-APB did not affect the total amount of EB1 in the shafts of GnRH neurons. However, EB1 localization to the actin cortex was significantly reduced after blocking IP3 receptors (Figure 4F, 1.1 ± 0.13 EB1 puncta/μm of cortical actin in *n* = 10 control neurons vs. 0.59 ± 0.05 EB1 puncta/μm of cortical actin in *n* = 12.2-APB treated neurons, *p* < 0.0006, *t*-test; Cohen's *d* = 1.75, *N* = 3 explants for both conditions). This result indicates that microtubule capture at the actin cortex is a physical interaction that is attenuated in the absence of calcium release.

## Discussion

The present results support a model for nuclear movement during neuronal migration whereby microtubules link to cortical actin draw the nucleus forward, flowing toward the growth cone, during calcium activity. Dissociation of microtubules from actin during calcium inhibition disrupts this process, resulting in microtubule translocation back toward the soma, possibly due to calcium-independent microtubule motor activity. In addition, our data show that during neuronal migration, microtubule capture at the moving actin cortex in the shaft transmits forces critical for nucleokinesis. These results reveal a fundamental mechanism of microtubule contribution to nucleokinesis as cells migrate to establish their proper neural circuits.

Genetic studies of tubulin subunits in neurological disorders have revealed that mutations in tubulin genes have severe effects on migration (Keays et al., 2007; Tischfield et al., 2011). However, mutations could affect any one of the many modes of migration used by the affected cells (neurite extension, multipolar movement, locomotion, or somal translocation) (Saillour et al., 2014). Thus, it is important to delineate cytoskeletal dynamics during neuronal migration in the unperturbed state, to better understand the etiology of the disease state.

Microtubules form a cage-like structure around the nucleus and extend into the leading process (Rivas and Hatten, 1995). As such, microtubules are well positioned to either pull or obstruct the nucleus. Microtubules are essential for leading process extension (Baudoin et al., 2008; Lysko et al., 2014). However, the contribution of microtubule dynamics to the movement of the soma during the nucleokinesis phase of neuronal migration is controversial. Movement of the centrosome, the structure where most microtubules are attached at their minus ends, precedes nucleokinesis in migrating cortical pyramidal neurons (Tsai et al., 2007). This observation is consistent with microtubules promoting migration by transmitting traction forces from the growth cone at the tip of the leading process (Asada and Sanada, 2010) and propulsion from actinomyosin contractions in the cell rear is halted when microtubules are artificially stabilized (Martini and Valdeolmillos, 2010). However, other work in cortical neurons suggests that leading process microtubules, combined with kinesin-5, form a molecular brake (Falnikar et al., 2011), consistent with data in migrating cerebellar granule neurons in which depolymerization of microtubules accelerated migration rates (He et al., 2010). Even in the same cell type, the centrosome sometimes leads the nucleus and sometimes trails (Yanagida et al., 2012). These studies suggest that mechanisms underlying migration are context-dependent and likely temporally modified. Thus, understanding neuronal migration will require discovering when and how microtubule mechanisms are engaged during neuronal migration.

Our results reveal a new mechanism engaged during the axophilic migration of GnRH neurons. We show that microtubule linkage to the dynamic cortical actin in the leading process shaft (Hutchins et al., 2013) promotes the forward movement of the nucleus. It is not known whether this is the same mechanism used to draw the centrosome forward in the radial migration of cortical neurons (Tsai et al., 2007), but appears to be independent of the mechanisms used in cortical interneurons and cerebellar granule cells that are either not affected or accelerate with microtubule depolymerization (He et al., 2010; Martini and Valdeolmillos, 2010; Falnikar et al., 2011). Although actin-dependent propulsion from the cell rear described in cortical interneurons and cerebellar granule cells (Martini and Valdeolmillos, 2010; Steinecke et al., 2014) does not discernably contribute to the microtubule/soma convergence observed in GnRH cells, it does correlate with ~30% of the forward movement that is unexplained by leading process actin dynamics (Hutchins et al., 2013). Other mechanisms involving iterative branching and retraction of the leading (or multipolar) process (Martini et al., 2009; Kitazawa et al., 2014) seem not to be utilized in GnRH neurons, which instead form long, mostly unbranched leading processes. Since many cells exhibit simple morphology when undergoing migration, the cytoskeletal dynamics described here for GnRH neurons may be a common developmental mechanism. As such, microtubule linkage to the dynamic cortical actin in the leading process shaft promoting forward movement of the nucleus, adds to a growing repertoire of microtubule-based cellular tools used by neurons to accelerate or slow their migration, along with microtubule braking (He et al., 2010; Falnikar et al., 2011) and +TIP-dependent leading process protrusion (Kholmanskikh et al., 2006).

### Conflict of interest statement

The authors declare that the research was conducted in the absence of any commercial or financial relationships that could be construed as a potential conflict of interest.

## Supplementary material

Movie 1 **Microtubule translocation and compression strongly predict movement of the nucleus when taken together**. Three-dimensional scatter plot of microtubule translocation (MTspeed) and microtubule/soma convergence (labeled MTcompression) rates vs. migration speed (soma speed). **(A–C)** Different views of the 3D scatter plot of these parameters with best-fit plane. Multiple regression *R*^2^ = 0.7696 (*R* = 0.88).

Movie 2 **Pseudocolored time-lapse video of labeled microtubules**. Arrows track microtubule bundles in each segment of the video (control vs. 2-APB treatment from the same cell). 1st half: microtubules advance as the soma moves forward (position of the cell border shown in an overlay). 2nd half: microtubules reverse toward the nucleus as movement stalls during 2-APB treatment.
